# Phenological tracking of migratory waterfowl to spring green-up derived from individual movement

**DOI:** 10.1186/s40462-026-00685-3

**Published:** 2026-07-27

**Authors:** Chengyi Liu, Peter M. van Bodegom, Fei Xu, Babak Naimi, Guangchun Lei, Jialin Lei, Yali Si

**Affiliations:** 1 Institute of Environmental Sciences, Leiden University, Leiden, the Netherlands; 2 Key Laboratory of the Three Gorges Reservoir Region’s Eco-Environment, Ministry of Education, Chongqing University, Chongqing, China; 3 Department of Biology, University of Utrecht, Utrecht, the Netherlands; 4 School of Ecology and Nature Conservation, Beijing Forestry University, Beijing, China; 5 Department of Earth System Science, Ministry of Education Field Research Station for East Asian Migratory Birds, Tsinghua University, Beijing, China

**Keywords:** Climate change, Bird migration, Onset of spring, Phenological asynchrony, Phenological sensitivity, Green-up synchrony, Phenological mismatch, Phenological response, Satellite tracking, East Asian-Australasian Flyway

## Abstract

**Background:**

Migratory birds are affected by phenological mismatches between migration timing and food availability under climate change. Birds can adjust their migration timing to some extent to track shifts in food availability, a phenomenon referred to as phenological sensitivity. Our understanding of species-specific phenological sensitivity and tracking in response to climate change remains limited, particularly because these processes have rarely been quantified using long-term individual movement data.

**Methods:**

In this study, we investigated the phenological tracking of migratory waterfowl by combining eight-year satellite tracking data of four species with spring green-up derived from remote sensing. Using Bayesian models and accounting for individual differences, we analyzed changes of bird arrival timing in relation to multi-year shifts in spring green-up, as well as responses to interannual variation.

**Results:**

Birds respond to shift in spring green-up with species-specific differences. Species with intermediate levels of individual variation (the greater white-fronted goose *Anser albifrons*) tend to align their migration timing better with both multi-year and interannual shifts in spring green-up, whereas species with excessive individual variation (the tundra bean goose *Anser serrirostris*) show limited degree of phenological tracking.

**Conclusions:**

Migratory waterfowl respond to both multi-year and interannual shifts in spring green-up, with individual variations influencing species-specific tracking. While intermediate individual variation may lead to more synchronous responses to climate change at the population level, excessive individual variations may reduce population-level synchrony and hinder adaptive response. Our findings advance the understanding of climate change impacts on migratory birds and their capacity to adapt to changing environments. Considering individual variation in phenological tracking is therefore essential when assessing species’ vulnerability to climate change.

**Supplementary Information:**

The online version contains supplementary material available at https://doi.org/10.1186/s40462-026-00685-3.

## Background

Phenology, the timing of seasonal life-history events of organisms, is influenced by the environmental conditions [1]. For example, the breeding phenology of animals is mainly affected by the phenology of their food supply [2]. Currently, a major driver of phenology shifts in numerous species is climate change, which affects different organisms differently [3]. Phenological mismatches can occur when predators and prey (or herbivores and plants) exhibit differential responses to climate change [4–6]. Phenological mismatch has been frequently documented and can negatively affect reproduction [7, 8], leading to population declines [9–11] and potentially altering ecosystem functioning [12, 13].

Migratory birds travel annually between low and high latitudes, relying heavily on seasonal food resources along their migration routes, which makes them particularly vulnerable to phenological mismatch [14–17]. Migratory birds can change their migration phenology in response to climate change, but their responses are often insufficient [18–23]. Previous studies reported that the response of birds to climate change (i.e., phenological sensitivity) could vary among species [20, 24–27]. This can be explained by specific traits, such as habitat types in use, trophic level and migratory strategy [28]. For example, birds that migrate slower, arrive earlier and wintering further north are found to be more sensitive and their migration timing is in general more synchronized with the conditions in higher-latitude areas [20]. Additionally, most bird species seem to align more closely to long-term average of green-up than with short-term current conditions [26].

Responses of birds to climate-driven phenological change are generally considered to arise through both phenotypic plasticity and microevolution [29], with phenotypic plasticity considered an important mechanism underlying phenological shifts [30–32]. While previous studies offer important insights, phenological tracking of migratory birds en route is often inferred from population-level migration timing derived from citizen science observation data, which are often subject to detection bias and data uncertainty [33, 34] and do not capture individual-level movement information. Because population-level plasticity emerges from the aggregation of individual responses to environmental cues, understanding variation in responsiveness among individuals may provide key insights into the mechanisms underlying population-level phenological adaptation.

Individual differences have been suggested to help migrants to synchronize their migration timing with spring green-up [35]. However, empirical evidence depicting fine-scale movement of individual birds along migration routes over extended period is still rare, resulting in limited understanding of how individual differences shape species’ responses to both long-term climate change and short-term variation of climate conditions across their migration corridor. The increasing availability of satellite tracking data offers great potential to study response mechanisms and phenological tracking while explicitly accounting for individual differences along migration routes.

In this study, we examined the phenological tracking of shifts in spring green-up along the migration routes of East Asian migratory waterfowl species using high-resolution movement of individual birds tracked from 2015 to 2022. This satellite tracking dataset contains 323 spring migration tracks of four herbivorous waterfowl species, including 132 tracks from greater white-fronted goose (*Anser albifrons*), 56 tracks from lesser white-fronted goose *(Anser erythropus*), 23 tracks from tundra swan (*Cygnus columbianus*), and 112 tracks from tundra bean goose (*Anser serrirostris*). We investigated two types of phenological tracking: Cohort multi-year phenological tracking quantifies how bird arrival timing covaries with spring green-up at multi-year temporal scales, whereas phenological sensitivity quantifies bird responses to interannual (year-to-year) variation in spring green-up. Both measures are based on individual-level behavior and estimate population-level effects.

We aim to answer the following questions: (1) To which degree do migratory waterfowl respond to multi-year and interannual shifts in spring green-up in core stopover and breeding areas, and (2) How does individual variation influence species’ phenological tracking? We link the migratory timing of these herbivorous birds along their migration routes during the study period with spring green-up, a surrogate of the onset of food availability [16, 17], derived from remote sensing data. We hypothesize that (1) birds respond to changes in spring green-up, but not sufficiently, because their responses may be constrained by information availability and physiological conditions; and (2) higher inter-individual variation in phenological responses may enhance synchrony between migratory timing and spring green-up, as such variation increases the range of responses available within a population to track spatiotemporal environmental variation.

## Methods

### Study area

The East Asian-Australasian Flyway is a critical migration corridor that supports a high diversity of migratory birds [36]. Yet, it is also a region marked by significant human-bird conflicts, such as habitat loss due to agriculture and aquaculture expansion, and an urgent need for effective conservation efforts [37–39]. Moreover, compared to Europe and North America, our understanding of migration ecology in East Asia remains limited [40], particularly regarding the responses of migratory birds to environmental change [41].

**Fig. 1:**
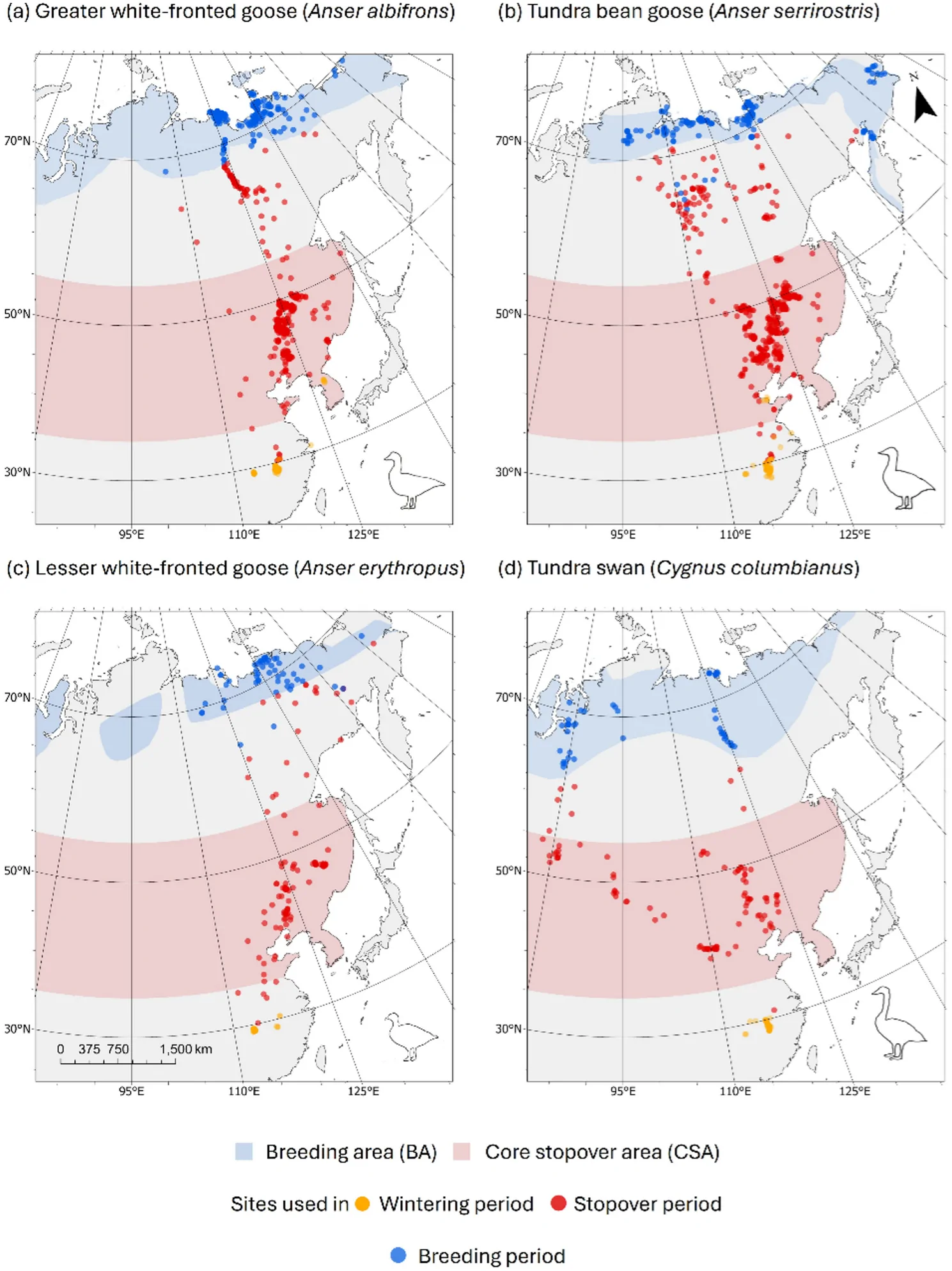
Sites used by East Asian waterfowl during spring migration from 2015 to 2022. Blue indicates breeding areas and red represents the core stopover area. Orange, red and blue dots are sites birds used during wintering, stopover and breeding periods, respectively. Few wintering sites located in the core stopover area are used by birds that winter at higher latitudes. Few sites used in breeding period but located near the breeding area are occupied by birds that spend summer outside the commonly defined breeding range. The north arrow indicates the true north direction at the center of the map

Figure 1 shows the migration corridors for the four East Asian migratory waterfowl species. The breeding areas for the greater white-fronted goose, lesser white-fronted goose and tundra swan were obtained from the Birdlife database [42]. For the tundra bean goose, we used the breeding area identified by [43], as the Birdlife database treats tundra bean goose as a subspecies of the bean goose (*Anser fabalis serrirostris*) and thus did not provide its breeding area separately. Wintering area was determined based on previous findings [40, 44] and visual inspection of tracking data. Birds predominantly wintered in Poyang Lake and Dongting Lake in the middle and lower Yangtze River Plain, whereas four individuals wintered in the northern China and one in the Korean Peninsula. The core stopover area was defined as the region between 35°N and 55°N latitude, which is used consistently and intensively by the birds across years [40].

### Satellite tracking of migratory waterfowl

Four herbivorous migratory waterfowl species, greater white-fronted goose, lesser white-fronted goose, tundra swan, and tundra bean goose, were tracked from 2015 to 2022. Birds were captured during the wintering season at Poyang Lake (29°N, 116°E) and Dongting Lake (29°N, 113°E) in the Yangtze River Floodplain, China. Birds were equipped with solar-powered GPS-GSM (Global Positioning System-Global System for Mobile Communications) neck loggers. The deployed devices weighed less than 3% of the body mass of different species to reduce the impacts [45], and detailed information of the devices can be found in Table A1-1, Supplementary Material 1. To minimize short-term behavioral effects, tagging was conducted at least five days before the start of migration for geese [44] and at least six weeks prior for swans [46]. The start of migration of geese and swans were referred to previous studies [47, 48]. GPS locations and corresponding time were recorded every 1–2 h and were resampled to 2–6 h to keep a consistent interval for each track. Detailed information about the bird capture can be found in [40] and [44].

### Identification of the migration pattern

We obtained a total of 323 spring migration tracks from 172 individuals collected between 2015 and 2022. We obtained 132 tracks from 64 individuals for greater white-fronted geese, 56 tracks from 43 individuals for lesser white-fronted geese, 23 tracks from 9 individuals for tundra swan, and 112 tracks from 56 individuals for tundra bean goose. A total of 39% of individuals had at least 2-year tracks. Detailed information on the number of tracks per species and per year can be found in Table A1-2, Supplementary Material 1.

We used the space-time permutation model in SaTScan software to identify the spatiotemporal clusters that represent sites where birds spend extended period of time during migration [49]. This model detects clusters of GPS locations of tracking data by scanning moving windows across space and time and testing whether the observed concentration of locations within a window is significantly higher than expected under a random use of space and time [40, 50]. More information about identifying stopover sites using SaTScan can be found in Supplementary Material 1, Section A1-2. We chose 50 km as the maximal spatial scanning window as the maximum foraging flight distance is generally smaller than this [40, 51–53]. Temporal scanning windows were set to be longer than two days considering birds need to stay in stopover sites for at least 48 h to settle and refuel [52]. Each identified space-time cluster includes information on the central location of the site, estimated circular range radius (range) and period of time (bird arrival and departure dates). All identified sites can be found in Supplementary Material 2. We categorized the sites into used during the wintering, stopover, or breeding periods, separately (Fig. 1).

### Estimation of bird arrival timing

We extracted the bird arrival date (AD) at each identified site for each track and calculated the multi-year change trend in bird arrival time. To assess the interannual change in bird arrival timing, we focused only on sites that were consistently used across multiple years during spring migration. For each track, we identified the first arrival site within the core stopover and the breeding area. Given some individuals were tracked over multiple years, we extracted a total of 482 first arrival sites from 323 tracks in total, from 172 individuals. The data summary can be found in Table 1. Not all tracked individuals successfully reached their breeding sites, resulting in fewer records for arrivals at the breeding sites.

Changes in arrival dates were determined by comparing the arrival dates of the same individual at pairs of first arrival sites located within 1° latitude across two consecutive years, treating these as the same site. In total, 129 pairs of arrival dates from 53 individuals were obtained (59 pairs for core stopover area and 70 for breeding area). A data summary can be found in Table 1. The number of pairs of arrival dates is limited to lesser white fronted geese (*N* = 8) and tundra swan (*N* = 8), and these two species were only included in the analysis examining the coherent multi-year phenological tracking.

**Table 1:** Data summary of first arrival sites and pairs of first arrival sites

Species	First arrival sites in core stopover areas	First arrival sites in breeding areas	Pairs in core stopover areas	Pairs in breeding areas
Greater white-fronted geese	129	70	27	20
Tundra bean geese	108	75	25	41
Lesser white-fronted geese	41	20	4	4
Tundra swan	20	19	3	5

For each pair of first arrival sites *i* and *j*, the change in arrival date (CAD) was calculated as:

1 $$CA{D_{ij}} = {\rm{ }}A{D_{i{\rm{ }}n}}-{\rm{ }}A{D_j}_{(n - 1)}$$

where AD_*i n*_ and AD_*j* (*n*−1)_ represent the arrival dates of the same individual at sites *i* and *j* in years *n* and *n*-1, respectively.

### Quantification of spring green-up

We used the spring green-up (SG) of herbaceous area as a proxy for the onset of food availability for the study species [16, 17]. We chose the Enhanced Vegetation Index (EVI) over the Normalized Difference Vegetation Index (NDVI) because it reduces background noise and minimizes saturation effects in areas with high biomass [54, 55]. We downloaded the 8-day 250-metre Moderate Resolution Imaging Spectroradiometer (MODIS) Terra surface reflectance product (MOD09Q1; v6.1) [56] and calculated EVI as:

2 $$\:\mathrm{E}\mathrm{V}\mathrm{I}=2.5\:\frac{\mathrm{N}-\mathrm{R}}{\mathrm{N}+2.4\mathrm{R}+1}$$

where N and R represent the surface reflectance in the near-infrared and the red bands, respectively [57].

We then smoothed the EVI time series using the Savitzky-Golay fitting method in TIMESAT 3.3 [58]. We set the half-window length to eight, corresponding to a 17-point moving window. This value is slightly smaller than the recommended guideline, reflecting a trade-off that prioritizes sensitivity to rapid temporal changes over stronger smoothing. Next, we calculated the rate of EVI change between each 8-day interval. The spring green-up was identified as the Julian day corresponding to the maximum rate of change. All calculations were executed in MATLAB R2022b [59]. We then extracted the herbaceous area from the spring green-up maps, including grassland, shrubland, flooded vegetation, and sparse vegetation, based on the 300 m Land cover data from Copernicus Climate Change Service, Climate Data Store [60]. We also compared the derived green-up date with the date when daily maximum temperature first exceeded 0 °C and kept only those green-up dates occurring after this threshold. The daily maximum temperature was derived from ERA5 hourly temperature data from Copernicus Climate Change Service, Climate Data Store (resolution is 0.5°×0.5°) [61]. For the core stopover area in the Northeast China Plain, we used the FROM-GLC Plus system based high-resolution land cover data (30 m resolution) [62] to exclude highly mixed areas, retaining only herbaceous grids with more than 50% of herbaceous cover [17]. Details of the calculations are provided in Supplementary Material 1, Section A1-3.

We used the median spring green-up date to represent the onset of vegetation growth at each first arrival site. The interannual change in the spring green-up (CiS) was calculated across consecutive years for each pair of first arrival sites *a* and *b*, assuming similar phenology dynamics within each site pair. CiS was defined as:

3 $$Ci{S_{ab}} = {\rm{ }}S{G_{a{\rm{ }}n}}-{\rm{ }}S{G_b}_{(n - 1)}$$

where SG_*a* n_ and SG_*b* (*n*−1)_ are the spring green-up at sites *a* and *b* in years *n* and *n*-1, respectively. All analyses were conducted in R-4.4.1 [63].

To evaluate this assumption, we calculated the difference in the green-up date between site *a* and site *b* in the first year of the pair. The mean difference was 1.77 days and the median difference was 0 days. The differences were not significantly different from zero (Wilcoxon signed-rank test, *p* > 0.05), indicating that green-up timing was generally well synchronized between paired sites within a given year.

### Coherent phenological tracking to multi-year shifts in spring green-up

To quantify temporal trends, we fitted Bayesian multivariate model with correlated residuals, relating spring green-up (SG) and bird arrival date (AD) to year for the core stopover and the breeding area, respectively. For each species and area, SG and AD were modelled as response variables within the joint framework and assumed to follow Gaussian distributions. To account for potential dependence between SG and AD and to estimate the phenological tracking from the posterior distributions, the two response variables were modeled jointly using a Bayesian multivariate model with correlated residuals. Individual identity was included as a random effect to account for potential repeated observations across years. Site was not included as a random effect because individuals exhibited minimal overlap in site use, with the mean pairwise overlap of first arrival sites within each year below 5%. Specifically, the component models related to SG and AD were specified as follows:

4 $${\rm{S}}{{\rm{G}}_{ij}} \sim N\left( {{\mu _{{\rm{S}}{{\rm{G}}_{ij}}}},\sigma _{{\rm{SG}}}^2} \right)$$

5 $${\rm{A}}{{\rm{D}}_{ij}} \sim N\left( {{\mu _{{\rm{A}}{{\rm{D}}_{ij}}}},\sigma _{{\rm{AD}}}^2} \right)$$

6 $$\eqalign{& {\mu _{{\rm{S}}{{\rm{G}}_{ij}}}} = {\alpha _{{\rm{SG}}}} + {\beta _{{\rm{SG}}}} \times {\rm{YEA}}{{\rm{R}}_{ij}} \cr & \,\,\,\, + {\gamma _{{\rm{SG}}}} \times {\rm{LATITUD}}{{\rm{E}}_{ij}} + {u_{{\rm{S}}{{\rm{G}}_j}}} \cr} $$

7 $$\eqalign{& {\mu _{{\rm{A}}{{\rm{D}}_{ij}}}} = {\alpha _{{\rm{AD}}}} + {\beta _{{\rm{AD}}}} \times {\rm{YEA}}{{\rm{R}}_{ij}} \cr & \,\,\, + {\gamma _{{\rm{AD}}}} \times {\rm{LATITUD}}{{\rm{E}}_{ij}} + {u_{{\rm{A}}{{\rm{D}}_j}}} \cr} $$

8 $$\:{u}_{{\mathrm{S}\mathrm{G}}_{j}}\sim\:N(0,\:{\sigma\:}_{{u}_{\mathrm{S}\mathrm{G}}}^{2})$$

9 $$\:{u}_{{\mathrm{A}\mathrm{D}}_{j}}\sim\:N(0,\:{\sigma\:}_{{u}_{\mathrm{A}\mathrm{D}}}^{2})$$

where *i* is the index of first arrival sites and *j* is the index of individuals, $\:{\mu\:}_{{\mathrm{S}\mathrm{G}}_{ij}}$ and $\:{\mu\:}_{{\mathrm{A}\mathrm{D}}_{ij}}$ are the expected value of SG and AD, $\:{\sigma\:}_{\mathrm{S}\mathrm{G}}^{2}$ and $\:{\sigma\:}_{\mathrm{A}\mathrm{D}}^{2}$ are the residual variances, assuming a Gaussian distribution, $\:{\alpha\:}_{\mathrm{S}\mathrm{G}}$ and $\:{\alpha\:}_{\mathrm{A}\mathrm{D}}$ are the intercepts, YEAR is the number of years since the first tracking year for the species, $\:{\beta\:}_{\mathrm{S}\mathrm{G}}$ and $\:{\beta\:}_{\mathrm{A}\mathrm{D}}$ are the slopes representing the rate of change in SG and AD, LATITUDE is the latitude of the first arrival site, $\:{\gamma\:}_{\mathrm{S}\mathrm{G}}$ and $\:{\gamma\:}_{\mathrm{A}\mathrm{D}}$ are the slopes, $\:{u}_{{\mathrm{S}\mathrm{G}}_{j}}$ and $\:{u}_{{\mathrm{A}\mathrm{D}}_{j}}$ represent the individual-level random intercept for individual *j*, assuming a Gaussian distribution with a mean of zero and variance $\:{\sigma\:}_{{u}_{\mathrm{S}\mathrm{G}}}^{2}$ and $\:{\sigma\:}_{{u}_{\mathrm{A}\mathrm{D}}}^{2}$.

We performed Markov Chain Monte Carlo sampling with four chains, each run for 10,000 warm-up iterations followed by 10,000 sampling iterations. We used weakly informative priors for all parameters and set the target average acceptance probability to 0.999. Model convergence was assessed via R-hat statistics, posterior distributions, and trace plots. All R-hat values were below 1.01, indicating good convergence without signs of non-convergence. Visual inspection of trace plots showed stable mixing and no apparent anomalies in the posterior distributions. In each model, the degree of coherent multi-year phenological tracking was quantified as a derived parameter by calculating the ratio of the temporal trends in arrival timing and spring green-up across posterior draws, where a value of 1 represents equal temporal changes in arrival timing and spring green-up. This ratio was computed only when both trends showed strong posterior support, defined as 95% credible intervals that did not overlap zero. The degree of coherent multi-year phenological tracking was summarized using the median and 95% credible intervals of the posterior ratio distribution.

To quantify individual variation in arrival timing, we calculated the annual standard deviation (SD) of arrival dates for each species within each area (core stopover area or breeding area). We then compared the annual variation in arrival timing between the greater white-fronted geese and tundra bean geese within each area using a Kruskal–Wallis test. This analysis was restricted to these two species because only they had sufficient tracking duration.

### Phenological sensitivity to interannual shifts in spring green-up

We constructed four Bayesian hierarchical models to quantify the phenological sensitivity of greater white-fronted geese and tundra bean geese in response to interannual shifts in spring green-up in core stopover and breeding areas. We excluded lesser white-fronted geese and tundra swan due to their low sample size. For each model, we set the change in arrival date (CAD) as the response variable. The model formulas are as follows:

10 $${\rm{CA}}{{\rm{D}}_{kj}} \sim N\left( {{\mu _{{\rm{CA}}{{\rm{D}}_{kj}}}},\sigma _{{\rm{CAD}}}^2} \right)$$

11 $$\eqalign{& {\mu _{{\rm{CA}}{{\rm{D}}_{kj}}}} = {\alpha _{{\rm{CAD}}}} + {\beta _{{\rm{CAD}}1}} \times {\rm{Ci}}{{\rm{S}}_{kj}} \cr & \,\, + {\beta _{{\rm{CAD}}2}} \times {\rm{DIFLA}}{{\rm{T}}_{kj}} + {b_j} \times {\rm{Ci}}{{\rm{S}}_{kj}} \cr} $$

12 $${b_j} \sim N\left( {0,\sigma _{{b_j}}^2} \right)$$

where *k* is the index of pairs of first arrival sites and *j* is the index of individuals, $\:{\mu\:}_{{\mathrm{C}\mathrm{A}\mathrm{D}}_{kj}}$ is the expected value of $\:{\mathrm{C}\mathrm{A}\mathrm{D}}_{kj}$, $\:{\sigma\:}_{\mathrm{C}\mathrm{A}\mathrm{D}}^{2}$ is the residual variance assuming a Gaussian distribution, $\:{\alpha\:}_{\mathrm{C}\mathrm{A}\mathrm{D}}$ is the intercept, $\:{\mathrm{C}\mathrm{i}\mathrm{S}}_{kj}$ is the change in spring green-up among the pairs of first arrival sites and $\:{\beta\:}_{\mathrm{C}\mathrm{A}\mathrm{D}1}$ is its estimated slope representing phenological sensitivity, $\:{\mathrm{D}\mathrm{I}\mathrm{F}\mathrm{L}\mathrm{A}\mathrm{T}}_{kj}$ is the latitudinal difference between the pair of first arrival sites, $\:{\beta\:}_{\mathrm{C}\mathrm{A}\mathrm{D}2}$ is the slope. $\:{b}_{j}$ is the individual-level random slope for individual *j* (one individual can have multiple pairs), assuming a Gaussian distribution with a mean of zero and variance $\:{\sigma\:}_{{b}_{j}}^{2}$.

We applied the same Bayesian model diagnostics as those of the previous models analyzing trends in spring green-up and bird arrival timing. Convergence diagnostics (R̂ < 1.01) indicated reliable sampling, with no divergent transitions observed. Considering the relatively lower sample size for these models, we report both 95% and 90% credible intervals for the estimated phenological sensitivity. All analyses were conducted using the *brms* package in R 4.4.1 [63, 64].

To facilitate interpretation of phenological sensitivity across species and areas, we plotted the individual variation in response to interannual shifts in green-up timing. Moreover, for each individual, we calculated the orthogonal distance to the 1:1 line (i.e., y = x) representing equal changes in bird arrival date and spring green-up. For individuals with multiple observations (≥ 2), the mean distance was used to summarize their overall response. We then quantified and compared the standard deviation of these individual-level distances for each species within each area, as a measure of individual variations in phenological response to interannual changes.

## Results

### Coherent phenological tracking to multi-year shifts in spring green-up

**Fig. 2:**
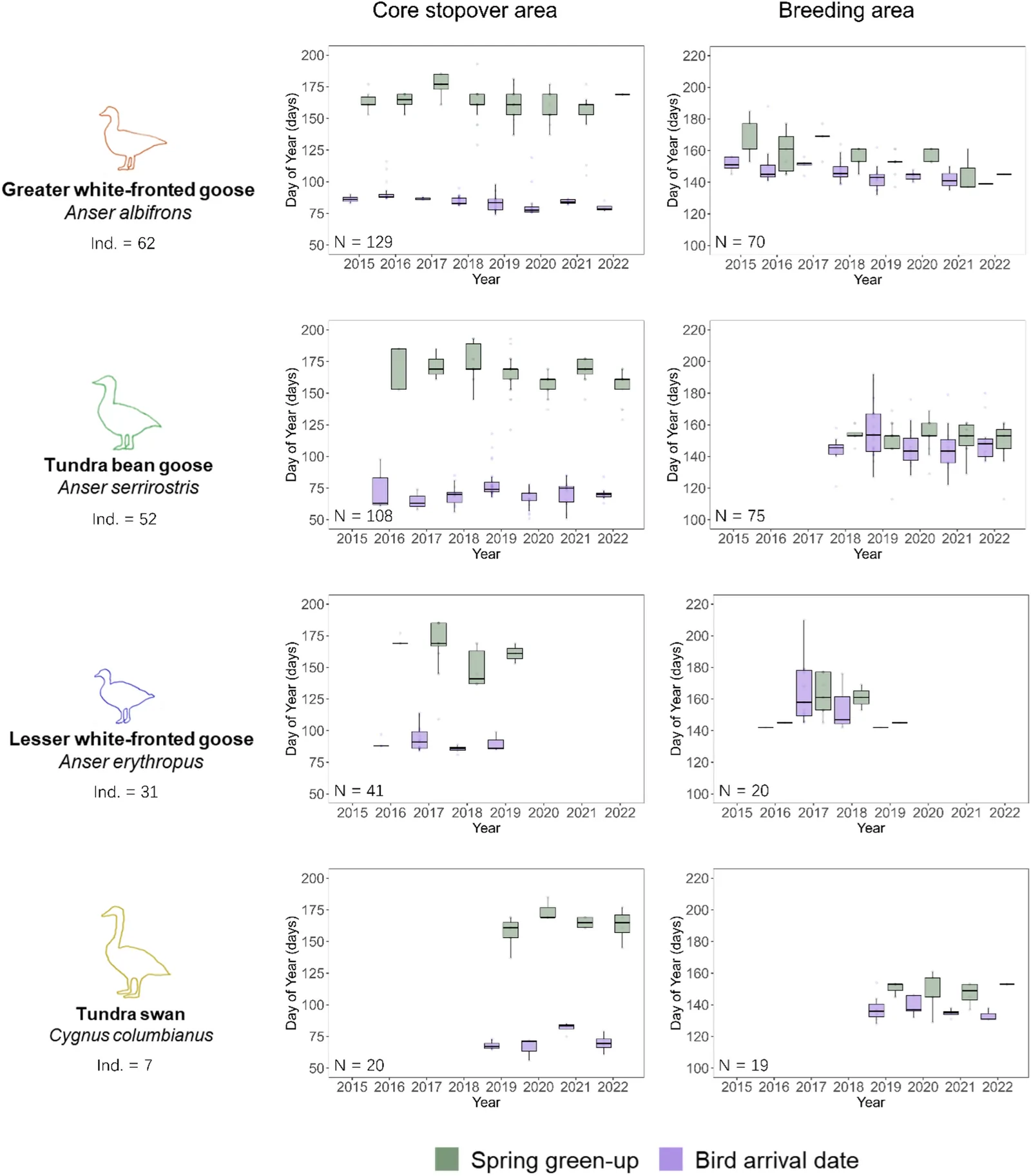
Arrival dates at core stopover and breeding areas for four East Asian migratory waterfowl species, along with the corresponding spring green-up from 2015 to 2022. Boxplots show the distributions of spring green-up dates (in green) and bird arrival dates (in purple). The box represents the interquartile range (from the first quartile, Q1, to the third quartile, Q3), and the horizontal line indicates the median

**Fig. 3:**
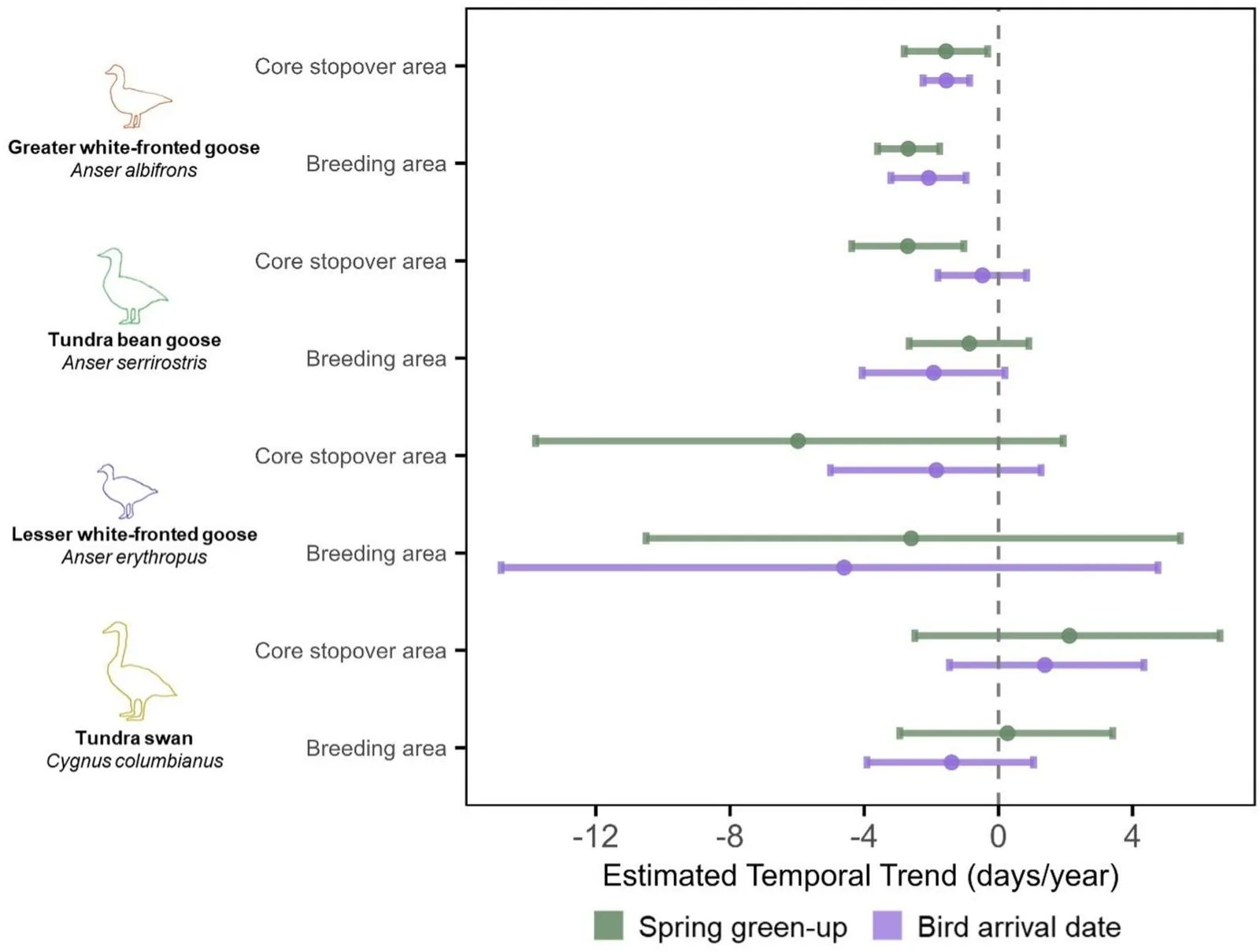
The multi-year trends of arrival dates and corresponding spring green-up from 2015 to 2022 at core stopover and breeding areas for East Asian migratory waterfowl. Points represent model-estimated trends, and horizontal lines indicate the 95% credible intervals. The vertical dashed gray line represents no trend

We found advancing trends in both spring green-up and bird arrival date over the study period (2015–2022) in both areas for great white-fronted goose (Fig. 2). In the core stopover area, the spring green-up advanced by 1.56 days per year (95% CI = − 2.81 - -0.32; Fig. 3; Supplementary Material 3. Table A3-1), while the arrival of birds advanced at a similar rate of 1.55 days per year (95% CI = -2.25 - -0.86; Fig. 3; Supplementary Material 3. Table A3-1). In the breeding area, the spring green-up advanced by 2.69 days per year (95% CI = -3.60 - -1.75; Fig. 3; Supplementary Material 3. Table A3-2), and birds advanced their arrival timing by 2.08 days per year (95% CI = -3.21 - -0.97; Fig. 3; Supplementary Material 3. Table A3-2). Based on these estimated trends, the degree of coherent multi-year phenological tracking for greater white-fronted goose was 0.98 (95% CI = 0.38–4.30) in the core stopover area and 0.77 (95% CI = 0.34–1.39) in the breeding area. We also found that spring advanced 2.70 days in the core stopover area for tundra bean geese (95% CI = -4.37 - -1.03; Fig. 3; Supplementary Material 3. Table A3-3), but birds did not advance their arrival time over time. No trends in spring green-up and bird arrival date were found for tundra bean geese in the breeding area, and neither for lesser-white fronted geese and tundra swan in stopover and breeding areas. In addition, we noticed that birds arrived much earlier than the spring green-up in the core stopover area. This is because they could use leftover seeds in agricultural land prior to the emergence of spring vegetation in this core stopover area. This pattern is not observed at sites in Russia where agricultural land is largely absent [17].

Regarding the comparison of the individual variation in migratory timing over the study period, the variation was significantly lower for greater white-fronted geese (mean SD in core stopover area: 5.3, mean SD in breeding area: 6.7) than for tundra bean geese (mean SD in core stopover area: 13.4, mean SD in breeding area: 9.4) in both areas (p-value < 0.05, Fig. 2).

### Phenological sensitivity to interannual shifts in spring green-up

**Fig. 4:**
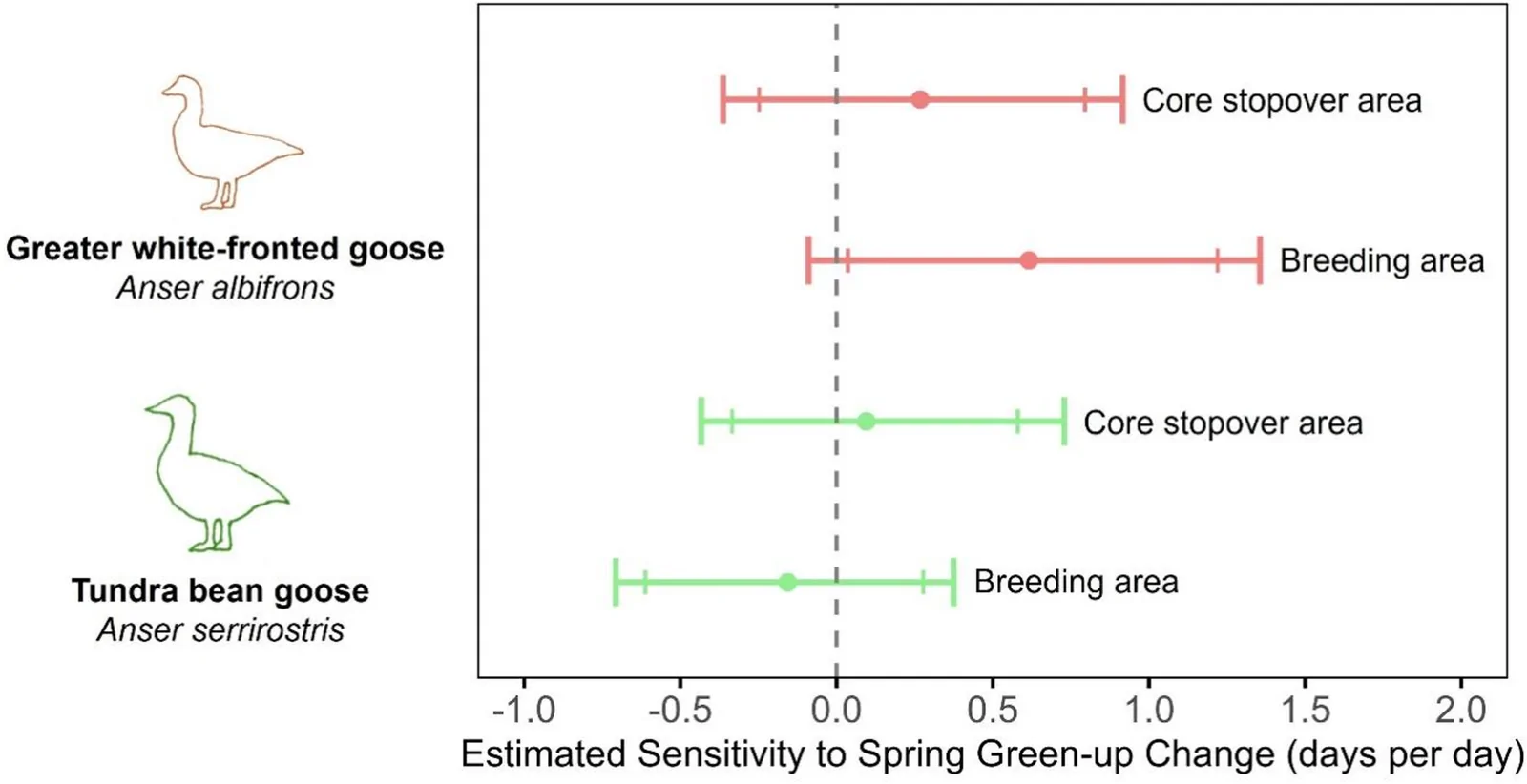
Phenological sensitivity to interannual shifts in spring green-up for migratory waterfowl in core stopover and breeding areas. Points represent the estimated sensitivity by the Bayesian model, horizontal lines indicate uncertainty, with longer range showing the 95% credible intervals and shorter range representing the 90% credible intervals. The vertical dashed gray line indicates a sensitivity estimate of zero

There is moderate to high probability that greater white-fronted geese are sensitive to interannual shifts in spring green-up in the breeding area, based on the 90% credible interval ($\:{\beta\:}_{\mathrm{C}\mathrm{A}\mathrm{D}1}$ = 0.62, 95% CI = -0.09–1.36, 90% CI = 0.04–1.22; Fig. 4; Supplementary Material 3. Table A3-10). No clear evidence of effect were found for the core stopover area for greater white-fronted geese ($\:{\beta\:}_{\mathrm{C}\mathrm{A}\mathrm{D}1}$ = 0.27, 95% CI = -0.36–0.92, 90% CI = -0.25–0.80; Fig. 4; Supplementary Material 3. Table A3-9) or for tundra bean geese in both areas (core stopover area: $\:{\beta\:}_{\mathrm{C}\mathrm{A}\mathrm{D}1}$ = 0.10, 95% CI = -0.43–0.73, 90% CI = -0.33–0.58; Fig. 4; Supplementary Material 3. Table A3-11; breeding area: $\:{\beta\:}_{\mathrm{C}\mathrm{A}\mathrm{D}1}$ = -0.16, 95% CI = -0.71–0.38, 90% CI = -0.61–0.28; Fig. 4; Supplementary Material 3. Table A3-12). The response of each individual to interannual changes in green-up can be found in Supplementary Material 1, Figure A-1.

**Fig. 5:**
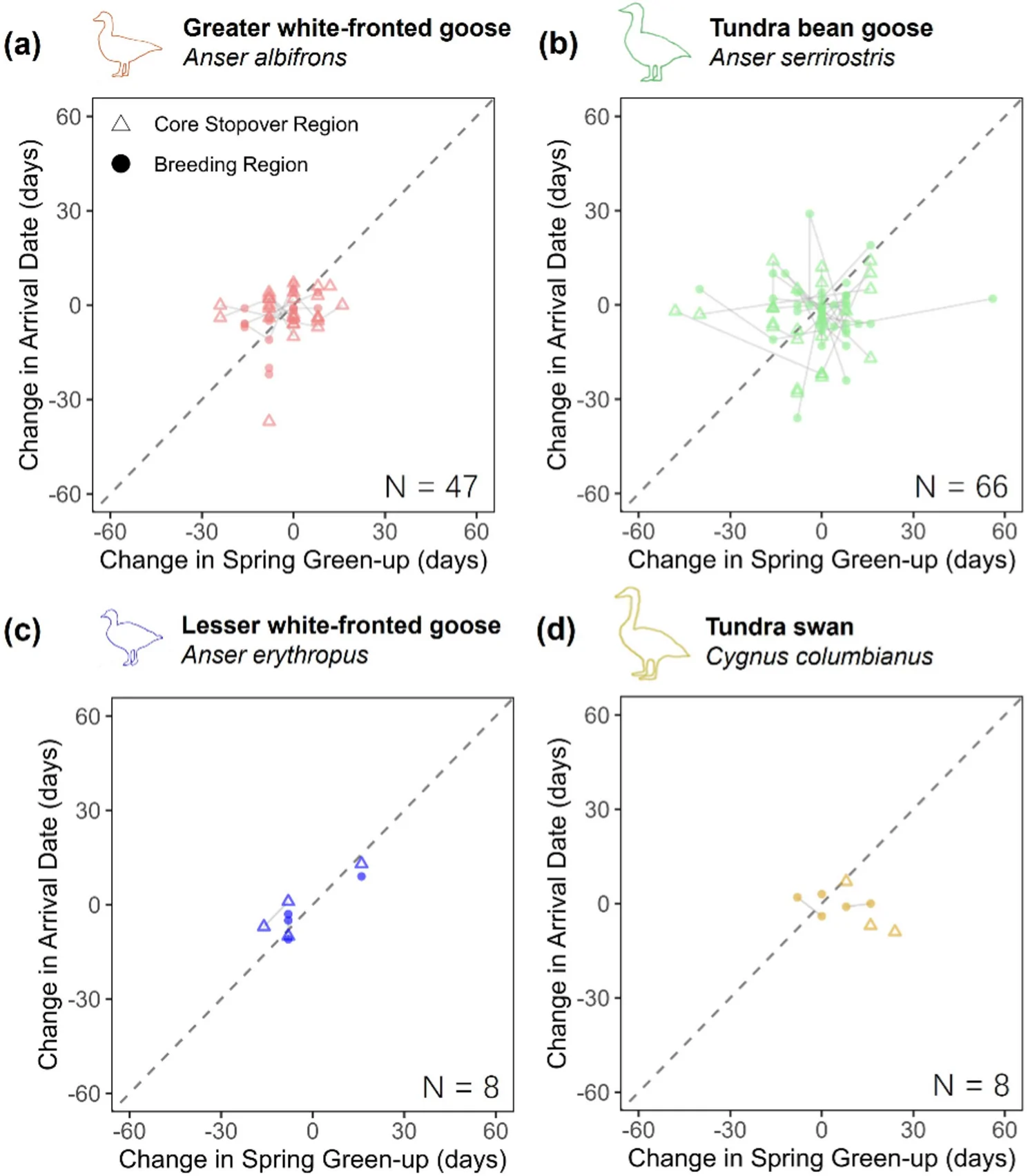
Individual responses to shifts in interannual spring green-up for migratory waterfowl. The grey dash line represents the equal changes in bird arrival date and spring green-up (y = x). Grey solid lines connecting points indicate data points from the same individual, which were tracked for at least three years

The individual responses to the interannual shifts in spring green-up timing were plotted for all species in two areas (Fig. 5). The individual variations (standard deviation) were relatively lower for lesser white-fronted geese (breeding area: 1.41 days, individual = 3; core stopover area: 2.68 days, individual = 3) and greater white-fronted geese (breeding area: 2.90 days, individual = 14; core stopover area: 4.46 days, individual = 19) than for tundra swan (breeding area: 3.37 days, individual = 3; core stopover area: 11.58 days, individual = 3) and tundra bean geese (breeding area: 5.54 days, individual = 16; core stopover area: 5.86 days, individual = 16).

## Discussion

This study investigates the phenological tracking of migratory waterfowl to multi-year and interannual shifts in spring green-up across species and areas based on individual movements in spring migration. We found that greater white-fronted geese show greater degree of phenological tracking by aligning their migration timing to both multi-year and interannual shifts in spring green-up whereas other species show limited sensitivity. Birds could align their arrival timing better in the breeding area than in the stopover area in responding to interannual shifts in spring green-up. Higher degree of phenological tracking is observed in species with moderate individual variation, whereas excessive variation leads to weaker phenological responses. Our findings suggest that moderate individual variation may lead to greater synchrony in responses to climate change at the population level, but excessive variation may hinder population-level synchrony and adaptation.

Greater white-fronted geese advanced their migration timing in response to the earlier spring green-up over the study period. Spring green-up advanced in both the core stopover area and the breeding area of greater white-fronted geese during the study period (2015–2022), with a relatively faster rate of advancement observed in the breeding area located at higher latitudes. This pattern is explained by the stronger warming trends at higher latitudes, which are known to accelerate the spring phenology [65]. We observed a moderate-level of individual variation in arrival timing which suggests that individual difference helps buffer against the shifts in spring green-up. While greater white-fronted geese adjusted their migration timing to match well with the multi-year shifts in vegetation green-up in both areas, the sensitivity is slightly higher in the core stopover area. This might be due to the climate variability in the wintering area is more consistent with that in the stopover area, than the variability between the stopover and breeding areas (quantified by ERA monthly temperature data [66], Supplementary Material 1, See Section A1-5). As a result, the timing of the spring green-up in the core stopover area may be better predicted based on climate conditions in the wintering area.

In contrast, spring green-up advanced in the core stopover area of tundra bean geese, but no advanced bird arrival timing is observed. This mismatch likely reflects differences in resource use compared with greater white-fronted geese. Although herbivorous waterfowl species often track newly emerged spring vegetation during migration [17, 67, 68], thereby optimizing their energy stores and enhancing breeding success [69], they differ in the extent to which they rely on this resource. Greater white-fronted geese depend strongly on newly emerging vegetation, particularly compared with the larger-bodied tundra bean goose [70, 71]. More generally, larger-bodied migratory waterfowl can exploit a broader range of food resources than smaller-bodied species [17, 72], consistent with the Jarman–Bell principle [73, 74]. Consequently, larger species may be less dependent on a single food cue and therefore less sensitive to shifts in spring green-up [16, 17].In addition, for lesser white-fronted geese and tundra swans, no clear trends were detected in either spring green-up or arrival timing across both areas. This might be caused by shorter tracking durations, limited sample sizes, and/or the absence of consistently directional changes in green-up timing.

In response to interannual shifts in spring green-up, greater white-fronted geese can better align their arrival timing in the breeding area than in the core stopover area. Birds have been found to exhibit higher phenological sensitivity at higher latitudes compared to lower latitudes [20, 27]. This may result from the necessity of arriving and breeding within a narrow temporal window in the Arctic [75]. Arriving too late at the Arctic breeding grounds may substantially reduce reproductive success [76–78]. In contrast, tundra bean geese did not synchronize their arrival timing with changes in spring green-up in either area. The different levels of variation in individual sensitivity may help explain this pattern. As illustrated by the standardized deviation in individual variation and in Fig. 4, greater white-fronted geese exhibit moderate individual variation, reflecting intermediate plasticity that allows effective alignment with resource phenology. Tundra bean geese, however, show a much broader range of individual responses, with many individuals poorly tracking green-up. Such excessive plasticity may reduce population-level synchrony and, illustrating how the balance of flexibility and consistency at the individual level can influence a species’ capacity to cope with shifting food phenology.

Although latitudinal differences were controlled for, when quantifying interannual change in spring green-up, local variation in spring green-up timing between paired sites may still introduce uncertainty. Future studies should incorporate measures of local habitat quality and bird staging duration to improve the accuracy of phenological tracking estimates under climate change. Moreover, non-climatic factors, such as anthropogenic disturbances and land-use change, may influence migration timing. Accounting for these drivers can provide a more comprehensive understanding of migratory birds’ responses. Nevertheless, considering individual variation provides new insights into phenological tracking. This approach allows individual exposure to spring green-up changes to be explicitly assessed, enable the tracking of responses over time, and accounts for both repeatability and inter-individual variability. Although species may share similar traits (e.g., migration distance), individual plasticity may be more informative than these coarser species-level traits and should be considered when explaining species-level phenological tracking.

## Conclusions

Migratory waterfowl respond to both multi-year and interannual shifts in spring green-up during spring migration, with responses varying among species. An intermediate level of individual variation imparts a more synchronous response to both multi-year and short-term shifts, whereas excessive individual variation can hinder adjustment to climate change in population level. This study provides important insights into the effect of individual variation on phenological tracking and enhances our understanding of species’ flexibility in changing environments. Individual variation should therefore be considered when assessing migratory species’ vulnerability to climate change.

## Supplementary Information

Below is the link to the electronic supplementary material.

Supplementary Material 1

Supplementary Material 2

Supplementary Material 3

Supplementary Material 4

## Data Availability

Tracking data is stored in in the Movebank Data repository (https://www.movebank.org), with the ID 52997422 and ID 731644623, and can be shared under request. Eight-day 250-metre Moderate Resolution Imaging Spectroradiometer (MODIS) Terra surface reflectance product (MOD09Q1; v6.1) is available via https://lpdaac.usgs.gov/products/mod09q1v061/ (DOI: 10.5067/MODIS/MOD09Q1.061). Bird breeding range is available from Birdlife DataZone (https://datazone.birdlife.org/) and Li et al. (2020), Wildfowl (Special Issue 6: 124–156). Global annual land cover data (300 m resolution) is available from Copernicus Climate Change Service, Climate Data Store (https://cds.climate.copernicus.eu/datasets/satellite-land-cover, DOI: 10.24381/cds.006f2c9a). ERA5 hourly temperature data can be accessed via https://cds.climate.copernicus.eu/datasets/reanalysis-era5-pressure-levels, DOI: 10.24381/cds.bd0915c6. ERA5 monthly temperature data can be accessed via https://cds.climate.copernicus.eu/datasets/reanalysis-era5-single-levels-monthly-means? tab=overview, DOI: 10.24381/cds.f17050d7. FROM-GLC (Finer Resolution Observation and Monitoring of Global Land Cover) is available from Yu et al. (2022) (DOI: 10.1080/15481603.2022.2096184). The list of all identified sites can be found in Supplementary Material 2 and all codes can be found in Supplementary Material 4.

## Abbreviations

CSA: Core stopover area
BA: Breeding area
AD: Arrival date
SG: Spring green-up
CAD: Change in arrival date
CiS: Change in the spring green-up
