# Supplementary material for: Phenological tracking of migratory waterfowl to spring green-up derived from individual movement
Source: Mov Ecol. 2026 Jul 27;14:75. doi: 10.1186/s40462-026-00685-3 (PMC13700227; doi:10.1186/s40462-026-00685-3)
Supplement: Supplementary file 1 [file 40462_2026_685_MOESM1_ESM.docx]

## Additional file 1. Supplementary Information on Methods

### Section A1-1. Summary of devices

**Table A1-1.** Summary of solar-powered GPS-GSM (Global Positioning System-Global System for Mobile Communications) neck loggers. The species names in the table are abbreviated as follows: GWFG: greater white-fronted geese, TUBG: tundra bean geese, LWFG: lesser white-fronted geese, TS: tundra swan.

| **Logger type** | **Logger name** | **Logger weight (g)** | **Tag type** | **Location error (m)** | **Company** | **Applied on (number of individuals)** | **Logger percentage body weight** |
| --- | --- | --- | --- | --- | --- | --- | --- |
| IBIS series neck loggers | IBIS series | 22 | Neckband | 20 | Ecotone Telemetry, Gdynia, Poland | GWFG (9), TUBG (2) | 0.88% (GWFG), 0.80% (TUBG) |
| HQNN series neck loggers | HQNN - large | 32 - 40 | Neckband | 0-5, 10-30, >100 | Hunan Global Messenger Technology Co. Ltd, Xiangtan, China | TS (9) | 0.50% - 0.63% |
|  | HQNN - medium | 26 |  |  |  | GWFG (42), TUBG (51) | 1.03% (GWFG), 0.94% (TUBG) |
|  | HQNN - small | 22 |  |  |  | LWFG (43) | 1.25% |
| DEBUT FLEX series neck loggers | DEBUT FLEX | 29.5 | Neckband | 5 | Druid Technology Co. Ltd, Chengdu, China | GWFG (13) | 1.18% |
| WT-300 series neck loggers | WT-300 | 72 | Neckband | < 20 | KoEco Inc., South Korea | TUBG (3) | 2.61% |

When calculating Logger percentage body weight, the average body mass of each species comes from EltonTraits [1].

**Table A1-2.** Summary of tracks per species per year.

| **SPECIES** | **Tracks in 2015** | **Tracks in 2016** | **Tracks in 2017** | **Tracks in 2018** | **Tracks in 2019** | **Tracks in 2020** | **Tracks in 2021** | **Tracks in 2022** |
| --- | --- | --- | --- | --- | --- | --- | --- | --- |
| GWFG | 6 | 16 | 10 | 36 | 31 | 18 | 11 | 4 |
| TUBG | 0 | 6 | 3 | 17 | 32 | 25 | 16 | 13 |
| LWFG | 0 | 11 | 33 | 8 | 3 | 1 | 0 | 0 |
| TS | 0 | 0 | 0 | 0 | 9 | 6 | 4 | 4 |

### Section A1-2. Details of SaTScan

The space–time permutation model was applied to detect spatiotemporal clustering patterns, using a cylindrical scanning window with a circular spatial base and a temporal height [2]. The scanning window expands from a single point up to predefined maximum spatial and temporal limits, which were determined based on the migration behavior of the target species.

In this study, we follow the settings from Si et al., 2018 [3]. The maximum spatial scanning radius was set to 50 km, consistent with previously reported maximum foraging flight distances of waterfowl and constrained to not exceed 50% of all GPS locations [4–6]. The maximum temporal scanning window was defined as 50% of the spring migration period. Detailed SaTScan parameter settings and data preprocessing procedures are provided in Additional file 4 (Codes).

### Section A1-3. Filtering of spring green-up data

After estimating the spring green-up, we applied three filtering steps to ensure data accuracy.

As the target waterfowl species forage in herbaceous habitats, we only kept the phenological data in herbaceous areas. To achieve this, we filtered phenological data by using the 300m land cover classification gridded maps from 2015 to 2022 derived from satellite observation datasets, from Copernicus Climate Change Service, Climate Data Store [7]. Phenological data corresponding to grassland, shrubland, flooded vegetation, and sparse vegetation were preserved for subsequent analysis.

We also applied temperature-based filtering to remove errors and invalid pixels. From ERA5 hourly 2 m temperature, we derived daily maximum temperature at 0.5° × 0.5° resolution and, for each year and area, identified the first day with temperature > 0 °C [8]. We then discarded any spring green-up dates earlier than this threshold and retained only those occurring later.

Furthermore, we observed that small farmland patches are widespread in the Northeast China Plain, the main part of the core stopover area. These farmlands are interspersed with herbaceous areas and cannot be fully excluded using the 300 m land cover dataset. As farmland exhibits different spring green-up dynamics, mixed pixels may bias phenological estimates [9]. To address this, we followed method from Fei et al., 2024 [10]. We extracted high-precision Northeast China Plain area land cover data with 30m resolution in the corresponding years [11]. The 2020 dataset was also applied to 2021 and 2022. We calculated the proportion of herbaceous area and non-herbaceous area for buffers with a radius of 150m around the center of each pixel of green-up data in the Northeast China Plain. Only raster pixels with herbaceous cover exceeding 50% were retained.

Filtering spring green-up data by landcover and temperature were carried out in ArcGIS 10.6 [12]. The codes for preprocessing temperature data and filtering green-up data can be found in Additional file 4.

### Section A1-4. Individual response to the interannual change in spring green up

**Figure A-1.** Individual-level responses in arrival date to interannual changes in spring green-up for greater white-fronted geese (GWFG) and tundra bean geese (TUBG) in core stopover areas (CSA) and breeding areas (BA). Black lines indicate model-predicted individual responses. Dashed lines indicate equal changes in bird arrival date and spring green-up (y = x).

### Section A1-5. Regional climate dynamics

To examine the climate dynamics across the wintering, core stopover, and breeding areas, we analyzed annual mean temperature (AMT) based on ERA5 monthly 2 m temperature data (2015–2022) [13].

We first calculated yearly AMT for each site, including first arrival sites in the core stopover and breeding area and the last wintering sites of each track. We obtained the AMT for each site across the 8 years. Then, for each area, we averaged AMT across all sites to derive the regional AMT for each year (see Table A1-3 and Figure A-1).

We further applied dynamic time warping (DTW) to assess climate similarity between areas. The DTW distance between wintering and core stopover areas (161.87) was smaller than that between core stopover and breeding area (245.79), suggesting stronger climatic correspondence between wintering and core stopover areas.

The codes of data treatments and calculating dynamic time warping can be found in can be found in Additional file 4.

**Table A1-3.** Regional annual mean temperature of wintering, core stopover and breeding areas.

| **Area** | **2015** | **2016** | **2017** | **2018** | **2019** | **2020** | **2021** | **2022** |
| --- | --- | --- | --- | --- | --- | --- | --- | --- |
| **WINTERING** | 18.05 | 18.40 | 18.44 | 18.58 | 18.57 | 18.37 | 18.84 | 18.74 |
| **CORE STOPOVER** | 7.56 | 6.89 | 7.58 | 7.33 | 7.92 | 7.61 | 7.23 | 6.99 |
| **BREEDING** | -10.97 | -10.28 | -10.35 | -10.11 | -9.73 | -7.99 | -10.73 | -9.90 |

**Figure A-2.** Dynamics of regional annual mean temperature in wintering, core stopover and breeding areas from 2015-2022.

### References

1. Wilman H, Belmaker J, Simpson J, De La Rosa C, Rivadeneira MM, Jetz W. EltonTraits 1.0: Species‐level foraging attributes of the world’s birds and mammals: Ecological Archives E095‐178. Ecology. 2014;95:2027–2027. https://doi.org/10.1890/13-1917.1

2. Kulldorff M, Heffernan R, Hartman J, Assunção R, Mostashari F. A Space–Time Permutation Scan Statistic for Disease Outbreak Detection. Blower SM, editor. PLoS Med. 2005;2:e59. https://doi.org/10.1371/journal.pmed.0020059

3. Si Y, Xu Y, Xu F, Li X, Zhang W, Wielstra B, et al. Spring migration patterns, habitat use, and stopover site protection status for two declining waterfowl species wintering in China as revealed by satellite tracking. Ecol Evol. 2018;8:6280–9. https://doi.org/10.1002/ece3.4174

4. Si Y, Skidmore AK, Wang T, De Boer WF, Toxopeus AG, Schlerf M, et al. Distribution of Barnacle Geese *Branta leucopsis* in Relation to Food Resources, Distance to Roosts, and the Location of Refuges. Ardea. 2011;99:217–26. https://doi.org/10.5253/078.099.0212

5. Ackerman JT, Takekawa JY, Orthmeyer DL, Fleskes JP, Yee JL, Kruse KL. Spatial Use by Wintering Greater White-Fronted Geese Relative to a Decade of Habitat Change in California’s Central Valley. J Wildl Manag. 2006;70:965–76. https://doi.org/10.2193/0022-541X(2006)70%5B965:SUBWGW%5D2.0.CO;2

6. Johnson WP, Schmidt PM, Taylor DP. Foraging flight distances of wintering ducks and geese: a review. Avian Conserv Ecol. 2014;9:art2. https://doi.org/10.5751/ACE-00683-090202

7. Copernicus Climate Change Service, Climate Data Store. Land cover classification gridded maps from 1992 to present derived from satellite observation [Internet]. 2019 [cited 2024 Mar 14]. https://doi.org/10.24381/cds.006f2c9a

8. Copernicus Climate Change Service, Climate Data Store. ERA5 hourly data on pressure levels from 1940 to present [Internet]. 2023 [cited 2024 Feb 17]. https://doi.org/10.24381/cds.bd0915c6

9. Chen X, Wang D, Chen J, Wang C, Shen M. The mixed pixel effect in land surface phenology: A simulation study. Remote Sens Environ. 2018;211:338–44. https://doi.org/10.1016/j.rse.2018.04.030

10. Xu F, Wu W, Wei J, Xin Q, Wielstra B, La Sorte FA, et al. Migratory herbivorous waterfowl track multiple resource waves during spring migration. Proc R Soc B Biol Sci. 2024;291:20241448.

11. Yu L, Du Z, Dong R, Zheng J, Tu Y, Chen X, et al. FROM-GLC Plus: toward near real-time and multi-resolution land cover mapping. GIScience Remote Sens. 2022;59:1026–47. https://doi.org/10.1080/15481603.2022.2096184

12. Esri. ArcGIS Desktop. Redlands, CA; 2018.

13. Copernicus Climate Change Service, Climate Data Store. ERA5 monthly averaged data on single levels from 1940 to present [Internet]. https://doi.org/10.24381/cds.f17050d7
