# Supplementary material for: Phenological tracking of migratory waterfowl to spring green-up derived from individual movement
Source: Mov Ecol. 2026 Jul 27;14:75. doi: 10.1186/s40462-026-00685-3 (PMC13700227; doi:10.1186/s40462-026-00685-3)
Supplement: Supplementary file 2 [file 40462_2026_685_MOESM3_ESM.docx]

## Additional file 3. Summary Tables of Models

**Table A3-1.** Summary of temporal trend estimates for spring green-up and bird arrival date at first arrival sites (FAS) within the core stopover area for greater white-fronted geese. As it is a joint model, the parameters from two sub models, spring green-up and bird arrival date, are named with the prefixes, SG_ and ARRIVALDATE_, respectively. YEAR represents the time (in years) from the start of tracking for the species to the year the FAS was recorded, LATITUDE represents the latitude of FAS, and sigma represents the residual standard deviation.

| **Parameter** | **Estimate** | **Est.Error** | **l-95% CI** | **u-95% CI** | **Rhat** | **Bulk_ESS** | **Tail_ESS** |
| --- | --- | --- | --- | --- | --- | --- | --- |
| **SG_Intercept** | 229.74 | 21.93 | 186.84 | 272.69 | 1.00 | 55117.42 | 30462.63 |
| **ARRIVALDATE_Intercept** | 80.44 | 11.92 | 57.12 | 104.10 | 1.00 | 49345.29 | 30690.15 |
| **SG_YEAR** | -1.56 | 0.63 | -2.81 | -0.32 | 1.00 | 60027.59 | 31066.14 |
| **SG_LATITUDE** | -1.41 | 0.50 | -2.38 | -0.44 | 1.00 | 54161.77 | 30882.34 |
| **ARRIVALDATE_YEAR** | -1.55 | 0.35 | -2.25 | -0.86 | 1.00 | 49119.90 | 32264.45 |
| **ARRIVALDATE_LATITUDE** | 0.23 | 0.27 | -0.31 | 0.75 | 1.00 | 49089.77 | 30810.91 |
| **sigma_SG** | 11.71 | 0.79 | 10.28 | 13.35 | 1.00 | 48087.68 | 29658.36 |
| **sigma_ARRIVALDATE** | 6.01 | 0.51 | 5.07 | 7.05 | 1.00 | 12472.21 | 24560.91 |

**Table A3-2.** Summary of temporal trend estimates for spring green-up and bird arrival date at first arrival sites within the breeding area for greater white-fronted geese.

| **Parameter** | **Estimate** | **Est.Error** | **l-95% CI** | **u-95% CI** | **Rhat** | **Bulk_ESS** | **Tail_ESS** |
| --- | --- | --- | --- | --- | --- | --- | --- |
| **SG_Intercept** | -67.74 | 31.10 | -128.56 | -5.97 | 1.00 | 46503.40 | 29809.05 |
| **ARRIVALDATE_Intercept** | 47.07 | 35.40 | -23.12 | 116.58 | 1.00 | 43167.15 | 29492.26 |
| **SG_YEAR** | -2.69 | 0.47 | -3.60 | -1.75 | 1.00 | 38524.05 | 28672.78 |
| **SG_LATITUDE** | 3.30 | 0.44 | 2.42 | 4.16 | 1.00 | 45544.01 | 29787.83 |
| **ARRIVALDATE_YEAR** | -2.08 | 0.57 | -3.21 | -0.97 | 1.00 | 31996.49 | 28603.44 |
| **ARRIVALDATE_LATITUDE** | 1.50 | 0.50 | 0.51 | 2.50 | 1.00 | 42276.37 | 29498.25 |
| **sigma_SG** | 5.84 | 0.57 | 4.82 | 7.05 | 1.00 | 28225.89 | 28800.03 |
| **sigma_ARRIVALDATE** | 6.28 | 0.95 | 4.55 | 8.08 | 1.00 | 3915.87 | 13584.38 |

**Table A3-3.** Summary of temporal trend estimates for spring green-up and bird arrival date at first arrival sites within the core stopover area for tundra bean geese.

| **Parameter** | **Estimate** | **Est.Error** | **l-95% CI** | **u-95% CI** | **Rhat** | **Bulk_ESS** | **Tail_ESS** |
| --- | --- | --- | --- | --- | --- | --- | --- |
| **SG_Intercept** | 223.01 | 22.14 | 180.00 | 266.31 | 1.00 | 65837.86 | 30214.32 |
| **ARRIVALDATE_Intercept** | 22.89 | 17.84 | -12.22 | 58.00 | 1.00 | 67271.38 | 29822.53 |
| **SG_YEAR** | -2.70 | 0.85 | -4.37 | -1.03 | 1.00 | 65518.26 | 30434.33 |
| **SG_LATITUDE** | -1.11 | 0.50 | -2.09 | -0.15 | 1.00 | 67121.53 | 30106.58 |
| **ARRIVALDATE_YEAR** | -0.47 | 0.67 | -1.80 | 0.84 | 1.00 | 68587.66 | 29954.00 |
| **ARRIVALDATE_LATITUDE** | 1.16 | 0.40 | 0.37 | 1.95 | 1.00 | 68636.56 | 29521.17 |
| **sigma_SG** | 12.66 | 0.94 | 10.96 | 14.66 | 1.00 | 54763.20 | 28963.35 |
| **sigma_ARRIVALDATE** | 9.99 | 0.77 | 8.58 | 11.61 | 1.00 | 43245.67 | 28993.57 |

**Table A3-4.** Summary of temporal trend estimates for spring green-up and bird arrival date at first arrival sites within the breeding area for tundra bean geese.

| **Parameter** | **Estimate** | **Est.Error** | **l-95% CI** | **u-95% CI** | **Rhat** | **Bulk_ESS** | **Tail_ESS** |
| --- | --- | --- | --- | --- | --- | --- | --- |
| **SG_Intercept** | 88.93 | 22.24 | 45.72 | 132.80 | 1.00 | 37780.90 | 28462.15 |
| **ARRIVALDATE_Intercept** | 81.04 | 44.30 | -1.12 | 172.57 | 1.00 | 13275.29 | 18227.14 |
| **SG_YEAR** | -0.87 | 0.91 | -2.66 | 0.91 | 1.00 | 40495.19 | 30302.01 |
| **SG_LATITUDE** | 0.94 | 0.32 | 0.31 | 1.56 | 1.00 | 35963.77 | 28179.74 |
| **ARRIVALDATE_YEAR** | -1.93 | 1.08 | -4.06 | 0.19 | 1.00 | 32179.11 | 29488.59 |
| **ARRIVALDATE_LATITUDE** | 1.08 | 0.63 | -0.23 | 2.25 | 1.00 | 13219.84 | 18612.68 |
| **sigma_SG** | 9.02 | 0.84 | 7.50 | 10.82 | 1.00 | 30024.21 | 28013.48 |
| **sigma_ARRIVALDATE** | 9.45 | 1.14 | 7.51 | 11.98 | 1.00 | 12797.71 | 20072.91 |

**Table A3-5.** Summary of temporal trend estimates for spring green-up and bird arrival date at first arrival sites within the core stopover area for lesser white-fronted geese.

| **Parameter** | **Estimate** | **Est.Error** | **l-95% CI** | **u-95% CI** | **Rhat** | **Bulk_ESS** | **Tail_ESS** |
| --- | --- | --- | --- | --- | --- | --- | --- |
| **SG_Intercept** | 198.96 | 45.35 | 110.32 | 288.88 | 1.00 | 33820.13 | 28010.33 |
| **ARRIVALDATE_Intercept** | 64.25 | 19.79 | 26.23 | 104.54 | 1.00 | 16319.40 | 26108.77 |
| **SG_YEAR** | -5.97 | 3.98 | -13.80 | 1.93 | 1.00 | 34018.61 | 29325.10 |
| **SG_LATITUDE** | -0.61 | 1.05 | -2.71 | 1.44 | 1.00 | 32209.03 | 27653.65 |
| **ARRIVALDATE_YEAR** | -1.85 | 1.58 | -5.01 | 1.27 | 1.00 | 29681.03 | 28437.44 |
| **ARRIVALDATE_LATITUDE** | 0.67 | 0.46 | -0.27 | 1.56 | 1.00 | 15510.96 | 25918.73 |
| **sigma_SG** | 16.49 | 2.54 | 11.87 | 21.97 | 1.00 | 16907.58 | 18660.42 |
| **sigma_ARRIVALDATE** | 5.82 | 1.57 | 3.34 | 9.12 | 1.00 | 3608.32 | 12398.77 |

**Table A3-6.** Summary of temporal trend estimates for spring green-up and bird arrival date at first arrival sites within the core stopover area for lesser white-fronted geese.

| **Parameter** | **Estimate** | **Est.Error** | **l-95% CI** | **u-95% CI** | **Rhat** | **Bulk_ESS** | **Tail_ESS** |
| --- | --- | --- | --- | --- | --- | --- | --- |
| **SG_Intercept** | 23.62 | 70.95 | -117.27 | 163.16 | 1.00 | 27464.64 | 28403.56 |
| **ARRIVALDATE_Intercept** | -52.55 | 103.65 | -225.96 | 185.08 | 1.00 | 6063.79 | 11775.96 |
| **SG_YEAR** | -2.60 | 4.02 | -10.50 | 5.42 | 1.00 | 32857.97 | 27099.11 |
| **SG_LATITUDE** | 2.04 | 1.02 | 0.03 | 4.06 | 1.00 | 27945.54 | 28462.07 |
| **ARRIVALDATE_YEAR** | -4.60 | 4.79 | -14.82 | 4.75 | 1.00 | 24664.73 | 12982.76 |
| **ARRIVALDATE_LATITUDE** | 3.15 | 1.48 | -0.26 | 5.62 | 1.00 | 6087.96 | 11560.30 |
| **sigma_SG** | 8.94 | 2.43 | 5.06 | 14.40 | 1.00 | 8597.88 | 19055.18 |
| **sigma_ARRIVALDATE** | 9.29 | 4.53 | 3.94 | 20.62 | 1.00 | 2309.67 | 6381.58 |

**Table A3-7.** Summary of temporal trend estimates for spring green-up and bird arrival date at first arrival sites within the core stopover area for tundra swans.

| **Parameter** | **Estimate** | **Est.Error** | **l-95% CI** | **u-95% CI** | **Rhat** | **Bulk_ESS** | **Tail_ESS** |
| --- | --- | --- | --- | --- | --- | --- | --- |
| **SG_Intercept** | 154.56 | 104.73 | -49.89 | 361.93 | 1.00 | 22651.54 | 27435.15 |
| **ARRIVALDATE_Intercept** | -74.36 | 64.16 | -202.19 | 53.36 | 1.00 | 26100.74 | 26860.11 |
| **SG_YEAR** | 2.12 | 2.29 | -2.49 | 6.60 | 1.00 | 37703.22 | 27525.66 |
| **SG_LATITUDE** | 0.16 | 2.57 | -4.92 | 5.17 | 1.00 | 22668.04 | 27760.33 |
| **ARRIVALDATE_YEAR** | 1.39 | 1.45 | -1.46 | 4.33 | 1.00 | 32152.00 | 25679.15 |
| **ARRIVALDATE_LATITUDE** | 3.52 | 1.58 | 0.38 | 6.66 | 1.00 | 26036.59 | 26848.83 |
| **sigma_SG** | 10.95 | 2.31 | 7.25 | 16.25 | 1.00 | 19000.60 | 26276.39 |
| **sigma_ARRIVALDATE** | 6.85 | 1.45 | 4.58 | 10.20 | 1.00 | 22556.60 | 26218.56 |

**Table A3-8.** Summary of temporal trend estimates for spring green-up and bird arrival date at first arrival sites within the core stopover area for tundra swans.

| **Parameter** | **Estimate** | **Est.Error** | **l-95% CI** | **u-95% CI** | **Rhat** | **Bulk_ESS** | **Tail_ESS** |
| --- | --- | --- | --- | --- | --- | --- | --- |
| **SG_Intercept** | 89.61 | 57.31 | -22.80 | 202.39 | 1.00 | 35694.03 | 26646.85 |
| **ARRIVALDATE_Intercept** | 65.61 | 45.20 | -23.69 | 155.75 | 1.00 | 36515.33 | 26499.38 |
| **SG_YEAR** | 0.27 | 1.60 | -2.94 | 3.41 | 1.00 | 35089.88 | 27614.21 |
| **SG_LATITUDE** | 0.93 | 0.87 | -0.78 | 2.65 | 1.00 | 35579.18 | 27141.55 |
| **ARRIVALDATE_YEAR** | -1.40 | 1.25 | -3.92 | 1.05 | 1.00 | 35673.68 | 26051.76 |
| **ARRIVALDATE_LATITUDE** | 1.12 | 0.69 | -0.26 | 2.48 | 1.00 | 36707.50 | 26508.87 |
| **sigma_SG** | 7.27 | 1.36 | 5.12 | 10.44 | 1.00 | 28260.85 | 26353.26 |
| **sigma_ARRIVALDATE** | 5.47 | 1.26 | 3.53 | 8.36 | 1.00 | 14375.68 | 24368.34 |

**Table A3-9.** Summary of phenological sensitivity estimates of interannual spring green-up change within the core stopover area for greater white-fronted geese. CiS represents the change in spring green-up within a pair of first arrival sites, DIFLAT represents the difference in latitude within a pair of first arrival sites, and sigma represents the residual standard deviation.

| **Parameter** | **Estimate** | **Est.Error** | **l-95% CI** | **u-95% CI** | **l-90% CI** | **u-90% CI** | **Rhat** | **Bulk_ESS** | **Tail_ESS** |
| --- | --- | --- | --- | --- | --- | --- | --- | --- | --- |
| **Intercept** | -2.65 | 1.47 | -5.52 | 0.29 | -5.04 | -0.22 | 1.00 | 29235.12 | 28371.85 |
| **CiS** | 0.27 | 0.32 | -0.36 | 0.92 | -0.25 | 0.80 | 1.00 | 13295.13 | 17144.94 |
| **DIFLAT** | 2.94 | 3.17 | -3.39 | 9.18 | -2.27 | 8.05 | 1.00 | 34477.46 | 24290.32 |
| **sigma** | 5.90 | 1.54 | 3.65 | 9.49 | 3.87 | 8.82 | 1.00 | 5518.87 | 12108.23 |

**Table A3-10.** Summary of phenological sensitivity estimates of interannual spring green-up change within the breeding area for greater white-fronted geese.

| **Parameter** | **Estimate** | **Est.Error** | **l-95% CI** | **u-95% CI** | **l-90% CI** | **u-90% CI** | **Rhat** | **Bulk_ESS** | **Tail_ESS** |
| --- | --- | --- | --- | --- | --- | --- | --- | --- | --- |
| **Intercept** | -0.27 | 1.47 | -3.46 | 2.44 | -2.81 | 1.98 | 1.00 | 22271.52 | 18696.58 |
| **CiS** | 0.62 | 0.36 | -0.09 | 1.36 | 0.04 | 1.22 | 1.00 | 10154.34 | 14041.17 |
| **DIFLAT** | 5.23 | 4.44 | -4.41 | 13.48 | -2.53 | 12.04 | 1.00 | 16129.26 | 16663.49 |
| **sigma** | 4.76 | 1.37 | 2.82 | 8.03 | 3.01 | 7.38 | 1.00 | 5660.42 | 11496.89 |

**Table A3-11.** Summary of phenological sensitivity estimates of interannual spring green-up change within the core stopover area for tundra bean geese.

| **Parameter** | **Estimate** | **Est.Error** | **l-95% CI** | **u-95% CI** | **l-90% CI** | **u-90% CI** | **Rhat** | **Bulk_ESS** | **Tail_ESS** |
| --- | --- | --- | --- | --- | --- | --- | --- | --- | --- |
| **Intercept** | -4.93 | 2.94 | -10.80 | 0.86 | -9.76 | -0.14 | 1.00 | 45315.71 | 28035.54 |
| **CiS** | 0.10 | 0.29 | -0.43 | 0.73 | -0.33 | 0.58 | 1.00 | 19008.48 | 14613.39 |
| **DIFLAT** | 1.58 | 8.20 | -14.55 | 17.80 | -11.87 | 14.97 | 1.00 | 36449.23 | 27264.10 |
| **sigma** | 13.28 | 2.21 | 9.70 | 18.33 | 10.12 | 17.28 | 1.00 | 27200.12 | 28035.51 |

**Table A3-12.** Summary of phenological sensitivity estimates of interannual spring green-up change within the breeding area for tundra bean geese.

| **Parameter** | **Estimate** | **Est.Error** | **l-95% CI** | **u-95% CI** | **l-90% CI** | **u-90% CI** | **Rhat** | **Bulk_ESS** | **Tail_ESS** |
| --- | --- | --- | --- | --- | --- | --- | --- | --- | --- |
| **Intercept** | -2.03 | 1.42 | -4.79 | 0.82 | -4.34 | 0.34 | 1.00 | 44993.25 | 29774.54 |
| **CiS** | -0.16 | 0.27 | -0.71 | 0.38 | -0.61 | 0.28 | 1.00 | 14461.14 | 19654.64 |
| **DIFLAT** | 3.68 | 5.88 | -8.01 | 15.32 | -5.92 | 13.33 | 1.00 | 29695.37 | 28457.77 |
| **sigma** | 8.84 | 1.26 | 6.75 | 11.66 | 7.03 | 11.11 | 1.00 | 15821.22 | 20299.07 |
